# Special Issue “Isotopic Techniques for Food Science”

**DOI:** 10.3390/molecules26010134

**Published:** 2020-12-30

**Authors:** Nives Ogrinc, Federica Camin

**Affiliations:** 1 Department of Environmental Sciences, Jožef Stefan Institute, Jamova 39, 1000 Ljubljana, Slovenia; 2 Jožef Stefan International Postgraduate School, Jamova 39, 1000 Ljubljana, Slovenia; 3 Department of Food Quality and Nutrition, Research and Innovation Centre, Fondazione Edmund Mach, via Mach 1, 38010 San Michele all’Adige, Italy; federica.camin@fmach.it; 4 Center Agriculture Food Environment (C3A), University of Trento, via Mach 1, 38010 San Michele all’Adige, Italy

Today, the analytical verification of food safety and quality together with authenticity and traceability plays a central role in food analysis. Over the past few years, there has been a renewed effort towards the development of new, rapid, and accurate methods for verifying both quality and safety. Among the exploitable techniques, stable isotope ratio analysis (SIRA) has gained increasing importance for determining the authenticity of foods of animal and plant origin for both producers and control agencies [1]. Furthermore, modern instruments can produce large complex datasets, which have, in parallel, led to the development of sophisticated chemometric methods for their evaluation, and the development of appropriate models with the ability to discriminate the authenticity and origin of food products. This Special Issue of *Molecules* seeks to explore emerging topics and trends in isotopic techniques and their application to food science—from instrumentation, measurement, and data analysis to novel applications. It contains a collection of eleven publications, which discuss the latest advances and challenges in this field.

Three of the published papers deal with milk and dairy products. For example, the study performed by Wijenayake et al. [2] uses multiple *δ*^2^H, *δ*^18^O, *δ*^13^C, and *δ*^15^N values from casein to verify the geographical origin of dairy products of New Zealand. The authors found that isotopes could distinguish milk products from different regions, except in cases where the feed type, farming environment, and agriculture practice were similar. Hamzić Gregorčič et al. [3] found that *δ*^18^O values were higher in sheep and goat’s milk when compared to cow milk, reflecting the isotopic composition of drinking water source and the effect of differences in the animal’s thermoregulatory physiologies. Potočnik et al. [4] focused on the determination of δ^13^C in milk fatty acids as a useful biomarker for determining the authenticity and regional traceability of milk. They found that the fatty acid composition differs significantly, depending more on year and season than on geographical region. The stable isotope composition of fatty acids also proved to be a better biomarker of metabolic transformation processes in ruminants than discriminating against the origin of milk. Further, the authors observed that milk from Alpine and Mediterranean regions has a higher percentage of ω-3 polyunsaturated fatty acids and conjugated linoleic acid, compounds that are known to have positive effects on human health. Thus, this milk is likely “healthier” than milk produced in other regions in Slovenia.

Three papers describe the development of new methods in food authenticity to support metrology. The aim of the study performed by Camin et al. [5] was to define standard deviations of repeatability (sr) and reproducibility (sR) for vinegar and balsamic vinegar stable isotope ratios of H (D/H), C (*δ*^13^C), and O (*δ*^18^O), in order to establish them as fully recognized official standards. Seven laboratories were involved in this international collaborative study. Perini et al. [6] investigated the effect that fermentation water has on stable isotopic D/H ratios of alcohol obtained from concentrated grape must. The results indicated that correction equations quoted in the official Office International de la Vigne et du Vin (OIV) method for the (D/H)_I_ and (D/H)_II_ values in ethanol from concentrated must are needed to obtain reliable results. The analysis of *δ*^18^O in the majority of cases should be sufficient for a reliable correction of the data. When the *δ*^18^O value of the diluted must is higher than −5‰, *δ*^2^H should also be analyzed. The study performed by Hamzić Gregorčič et al. [3] uses *δ*^18^O values in lactose to identify the adulteration of milk and detection of water addition. The results are promising and indicate that *δ*^18^O values in lactose can be used as an internal standard in the detection of the illegal watering of milk (>7%).

Martinelli et al. [7] provided a baseline assessment of the isotope composition of carbon and nitrogen in food and beverages available on the Brazilian market. Their study included 1245 foods of animal and plant origin and 374 beverages. The data indicate that significant amounts of C-C4 plants (cane, maize) are present in the Brazilian food system, which is reflected in the more positive *δ*^13^C tissue values in the national population. Thus, these data can be used not only in food authenticity, but also in human anthropology studies.

This special issue also includes several papers that use stable isotope ratios, elemental profiles, and fatty acid composition to assure the authenticity and origin of high-quality products typical of the national identities of Mediterranean countries such as Moroccan argan oil [8], Portuguese meat products from Iberian black pigs [9], and Slovenian truffles [10]. These studies form part of the REALMed project “Pursuing authenticity and valorization of Mediterranean traditional products” (ARIMNET 2 call 2016) that aimed to provide reliable technological tools for authentication and quality assessment and make these tools widely available to stakeholders and consumers and promote traditional knowledge and the sustainable development of local economies [11]. The study performed on cosmetic argan oil shows the feasibility of implementing authenticity criteria for argan oils by including limit values for trans-fatty acids and the ability to discern provenance using fatty acid profiling [8]. The research performed on truffles indicated that elemental and stable isotope composition together with multivariate statistical analysis classified truffles according to species and geographical origin [10]. Cross-validation resulted in a 74% correct classification rate for determining the species and a 77% correct classification rate for geographical origin discrimination. The critical parameters for geographical origin discriminations were Sr, Ba, V, Pb, Ni, Cr, Ba/Ca, and Sr/Ca ratios, while, from stable isotopes, *δ*^18^O and *δ*^13^C values are the most important.

In the study performed by Alegria et al. [9], acorns of the two main *Quercus* species of the Portuguese *Montado*, a main feed of the renowned black Iberian pig was investigated, and the authors studied how morphological parameters and isotopic signature of these two species respond across an aridity gradient in the Alentejo region, Portugal. Although there was no difference in *δ*^15^N and *δ*^13^C values between the two species, multivariate analysis and geospatial data analysis clearly show the influence of long-term climatic variables on the acorn nitrogen isotope signature. The *δ*^15^N values are distributed according to the aridity index map, where higher *δ*^15^N values are found in more dry areas. From this data, the authors were able to generate an isoscape model, which is a spatially georeferenced representation of the distribution of isotopic compositions (generally of light elements). This approach can provide a cost-efficient tool for determination of provenience of high-quality food [12].

The use of stable isotopes of heavier elements such as strontium (Sr) and lead (Pb) can further help to trace the geochemical fingerprinting of a particular region to its food products. In the study performed by Lancellotti et al. [13], the lead isotope signature was tested as a geographical tracer for wine production and particularly for the Lambrusco Protected Designation of Origin (PDO) wines of the province of Modena (Italy). The results indicate the multiple lead sources present in bottled wine; however, none of them corresponds to the one present in soil, showing that anthropogenic sources of lead probably interfere in the soil–wine system. Conversely, ^87^Sr/^86^Sr values in truffles indicate that truffles reflect the local geochemistry of the environment in which they grow [10]. The ^87^Sr/^86^Sr of truffles appeared initially to be controlled by the carbonate fraction of soil, which makes Sr values useful for determining provenance in areas with and without limestone. The study also highlights uncertainties and site-specific challenges associated with determining Sr stable isotopes in truffles, since the mycorrhizal relationships with trees and mineral weathering are equally complex, often involving varying combinations of Sr sources and isotopic signatures, thereby making the determination of geographical origin an even greater challenge.

Further, Miklavčič Višnjevec and Schwarzkopf [14] propose that bioactive compounds such as phenolic compounds found in typical Mediterranean plants relative to genetic and environmental factors could be used in food authentication control. However, the authors conclude that many of the Mediterranean plants found in Istria that are rich in phenolic compound content and have an essential role in human nutrition are understudied and require further investigation.

Some of the manuscripts [3,4,8,10] presented their raw data in the supplementary material supporting the open-access of research data. Much of this data is also in the *e*-component of different projects such as METROFOOD-RI research infrastructure and FNS-Cloud. It is expected that such platforms will integrate various data sources, and services for comparing and making interoperable food data and will be accessible to the user community (Europe and beyond). We are sure that this Special Issue will be a valuable resource for those interested in the use of isotopes in food science for authenticity and origin verification.
